# Effects of Tapered-Strut Design on Fatigue Life Enhancement of Peripheral Stents

**DOI:** 10.3390/bioengineering10040443

**Published:** 2023-04-02

**Authors:** Li-Han Lin, Kuang-Lei Ho, Yu-Min Jian, Kuang-Hsing Chiang, Hao-Ming Hsiao

**Affiliations:** 1 Department of Mechanical Engineering, National Taiwan University, Taipei City 106, Taiwan; 2 Division of Cardiology and Cardiovascular Research Center, Department of Internal Medicine, Taipei Medical University Hospital, Taipei City 110, Taiwan

**Keywords:** peripheral artery disease, nitinol stent, tapered-strut design, stent fatigue life, goodman life analysis

## Abstract

Peripheral stent could fracture from cyclic loadings as a result of our blood pressures or daily activities. Fatigue performance has therefore become a key issue for peripheral stent design. A simple yet powerful tapered-strut design concept for fatigue life enhancement was investigated. This concept is to move the stress concentration away from the crown and re-distribute the stresses along the strut by narrowing the strut geometry. Finite element analysis was performed to evaluate the stent fatigue performance under various conditions consistent with the current clinical practice. Thirty stent prototypes were manufactured in-house by laser with a series of post-laser treatments, followed by the validation of bench fatigue tests for proof of concept. FEA simulation results show that the fatigue safety factor of the 40% tapered-strut design increased by 4.2 times that of a standard counterpart, which was validated by bench tests with 6.6-times and 5.9-times fatigue enhancement at room temperature and body temperature, respectively. Bench fatigue test results agreed very well with the increasing trend predicted by FEA simulation. The effects of the tapered-strut design were significant and could be considered as an option for fatigue optimization of future stent designs.

## 1. Introduction

Peripheral artery disease (PAD) mainly results from atherosclerosis which induces arterial blockage and limits the blood flow to the head, organs, and lower limbs. It often causes discomfort, pain, or even serious issues such as heart disease and ischemic stroke. In 2015, peripheral artery disease affected 155 million people around the world, among which 52,500 individuals died as a consequence [1,2].

Interventional cardiology has evolved enormously since its introduction in 1990s and become one of the most popular surgical procedures in contemporary clinical practice. It has seen waves of innovations to improve its treatments of various cardiovascular and neurovascular diseases. With this new technology advancement, intravascular stent has expanded its applications to many of our vascular system including peripheral arteries and other non-vascular applications such as biliary, urinary, gastrointestinal tracts, just to name a few.

**Figure 1:**
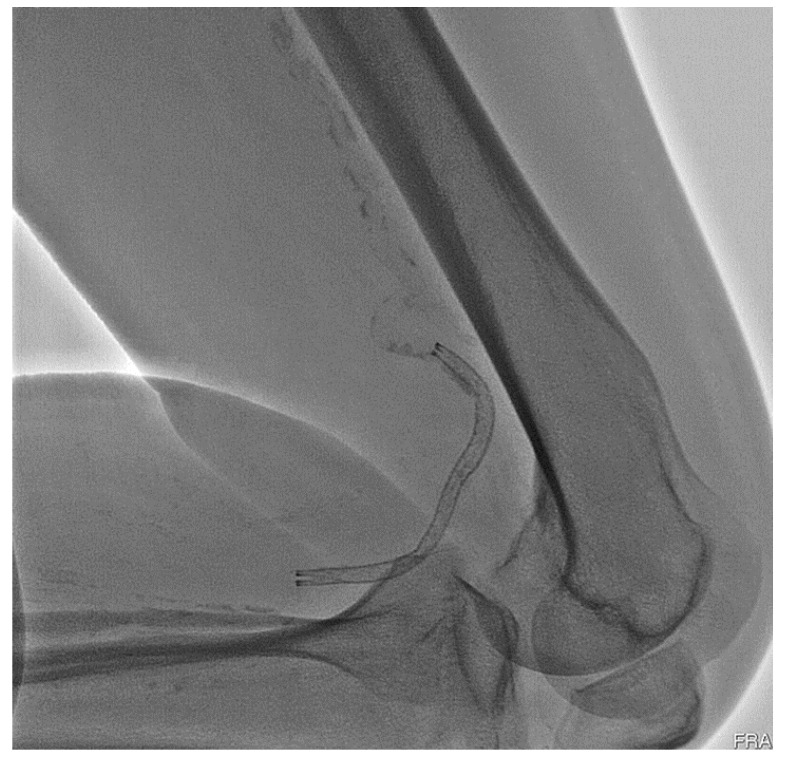
Deformed shape of a peripheral stent inside a bent patient knee.

However, peripheral stent could fracture from cyclic loadings due to blood pressures and our body activities after surgery. Such repeated movements continue to oscillate the stent, leading to the risk of fatigue fracture which may cause radial support loss to the arterial wall, in-stent restenosis, thrombus formation, or arterial perforation by sharp strut protrusion [3,4,5]. Partial or complete stent fatigue fractures have been reported in the cases of lower limbs with high fracture rates [6,7,8,9,10]. Peripheral stent fatigue fracture is a known complication of peripheral intervention and is related to increased re-occlusion risk of the treated artery. It has attracted many attentions in the past decades and, therefore, stent fatigue life is a key performance issue for many contemporary peripheral stent designs. Figure 1 shows the deformed shape of an implanted peripheral stent subjected to complex loading conditions when a patient bent his knee.

In the medical device industry, nitinol alloy (Ni-Ti) has gained popularity due to its excellent super-elasticity and fatigue resistance. Peripheral stent is particularly the most popular application of the nitinol alloy and demonstrates excellent clinical outcome [11,12,13,14]. It is super-elastic and crush-resistant, exerts a gentle chronic outward force (COF), and is more compatible to human physiology in peripheral indications. When deployed, the stent deformation varies from section to section due to the complicated loading modes of our body movements. In certain sections, the stent is in the pure austenitic phase, whereas in other sections, it could be partially transformed to the martensite phase where it cycles between the upper plateau and lower plateau of the nitinol stress–strain curve. As a result, the developed stresses inside the stent are complicated, leading to higher risk of fatigue fracture and thus unsuccessful treatment [15,16,17].

Past studies have addressed the stress analysis of the nitinol stent and provided good examples of the stent design optimization process [18,19,20,21,22,23,24]; however, only a handful of papers have focused on the fatigue life analysis of the nitinol stent. A stent-artery interaction model was developed earlier to analyze the effects of bending and compressive loadings on the stent fatigue performance. The conclusions showed that both types of loadings play important roles in stent fatigue fracture, depending on the implantation location within the vascular tree [25]. Meoli et al. analyzed the stent fatigue performance subjected to cyclic bending and compression in the fully deployed configurations [26,27]. Lei et al. showed that the stent-to-artery oversize ratio should be considered in the fatigue analysis of a stent [28]. Azaouzi et al. developed a simulation tactic to optimize the stent fatigue performance, which was built on the foundation of the response surface method in combination with Kriging interpolation [29]. Alaimo et al. proposed an optimization scheme focusing on improving the fatigue performance through the modification of the strut profile. Their computational model relied on the non-linear finite element analysis with an algorithm based on Kriging response surfaces [30].

In this study, a simple yet powerful tapered-strut design concept that increases the stent fatigue life [31,32,33] was investigated numerically and experimentally. Finite element analysis was performed to assess the mechanical integrity and fatigue resistance of this unique tapered-strut design under conditions consistent with the current practice. Bench fatigue tests were conducted to validate this stent design concept. The conclusions of this study could provide useful insights to the design optimization of future peripheral stents for their fatigue life enhancement.

## 2. Materials and Methods

### 2.1. Tapered-Strut Stent Design

**Figure 2:**
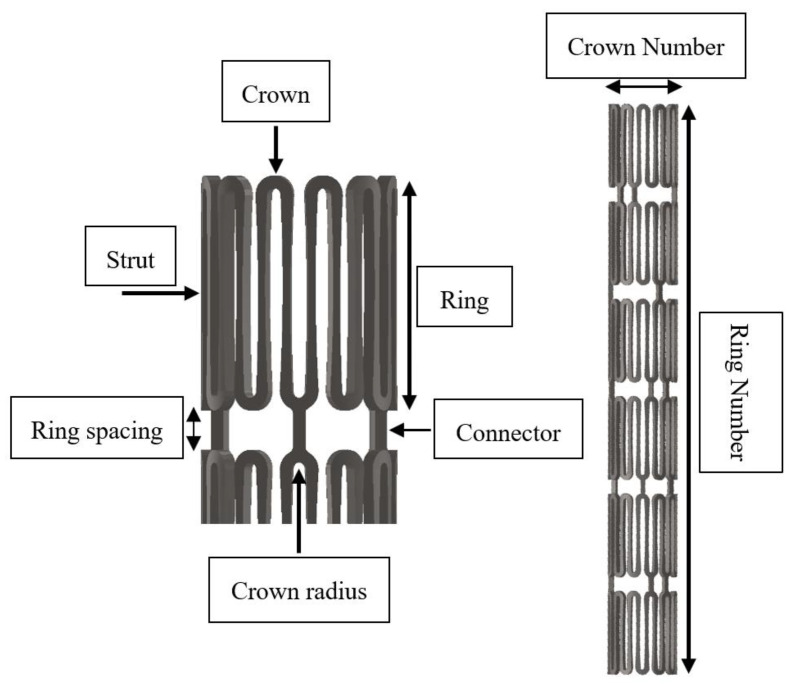
Illustration of important design parameters of an intravascular stent.

The stent design pattern consists of a series of rings bridged by a few connectors. The specific design may change, depending on the intended use and the anatomical location of the stent. Figure 2 illustrates the several important design parameters of an intravascular stent. The major parameter is the crown, as it dictates very important clinical attributes such as acute fracture and chronic fatigue failure. The strut thickness also plays a key role in the development of post-stenting re-narrowing, whereas the strut width and length control two important clinical attributes, namely, radial strength and vessel scaffolding, respectively [24,34].

**Figure 3:**
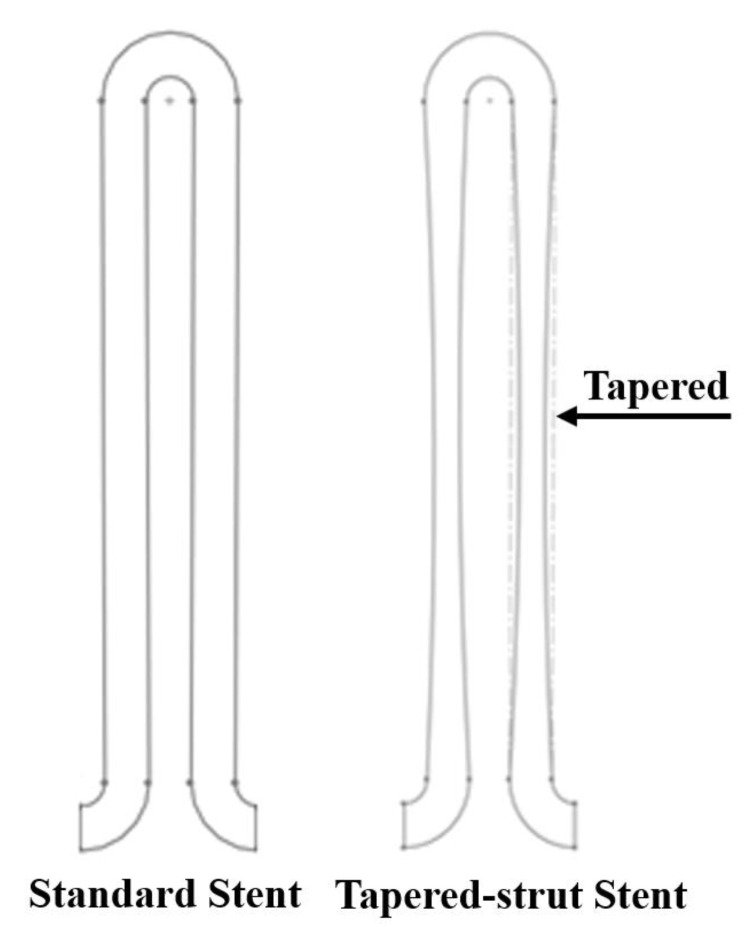
Tapered-strut stent design concept.

The tapered-strut design concept is to move the stress concentration away from the crown and re-distribute the stresses along the strut by tapering the strut width at the midpoint. This slight design tweak is able to reduce the loading burden on the crown, allow more efficient energy distribution, and thus significantly improve the stent fatigue life. To build this tapered-strut design, a parabolic curve was applied to connect two opposing semicircular crowns and made tangent to the crown arc at the same time, which thus formed the tapered-strut stent design, as shown in Figure 3. The midpoint of the strut was tapered from 100% to 60% (0 to 40% width reduction) of its original width for investigation. This slight design tweak does not change other clinical attributes such as stent profile, scaffolding, foreshortening, and has many advantages over other conventional fatigue-enhanced design methodologies using larger crown radii (larger profile) or elongated strut lengths (less scaffolding).

### 2.2. Finite Element Models

Finite element analysis (FEA) is a computational simulation technology used in the engineering field to predict the behavior of complex structures and components including stents under different load conditions. It serves as an efficient tool for stent designs to investigate the mechanical integrity and mutual interaction at the heterogeneous interface among various parts such as balloon, stent, plaque, and artery. This gives great insights to many aspects of the stent clinical attributes, which could eventually lead to much better clinical outcome [35,36,37,38,39].

The manufacturing of a nitinol stent involves an alternating series of expansions and heat treatments as the stent is gradually shaped into its target configuration. The repeated heat treatment after each expansion relieves all residual stresses in a stent by altering the microstructure of the material, allowing the stent to be further expanded to the next size without stent fractures. After deployment, the stent is subjected to all types of loadings and may survive or fail during its service life. Here the FEA analysis was performed using ABAQUS software (Dassault Syste`mes Simulia Corp., Providence, RI, USA) to assess the mechanical integrity and fatigue performance of a stent subjected to loading conditions encountered from manufacturing to implementation, including multiple stent expansions and their subsequent heat treatments, stent crimping inside a catheter, stent deployment into an artery, and stent fatigue life analysis under systolic/diastolic blood pressures.

#### 2.2.1. Material Properties

Nitinol alloys have quickly become the material of choice for self-expanding stents, grafts, heart valves, filters, plugs, and many medical devices for interventional procedures. The nitinol hypotube size used in this paper for making peripheral stents was 2.0 mm OD (Outer Diameter), extruded by Minitubes (Grenoble, France). The hypotube material was composed of mainly 54 to 57% nickel and 43 to 46% titanium by weight percent, with fractions of other compositions such as cobalt, iron, carbon, nioium, oxygen, and nitrogen. It has the Young’s modulus of 60 GPa, ultimate tensile strength of at least 1000 MPa, and ultimate strain of 10%. Nitinol has a unique stress–strain behavior with a hysteresis loop due to its capability to “remember” its trained shape. The hysteresis loop represents the energy dissipated during the loading–unloading process. The shape of the hysteresis loop depends on the specific chemical composition and processing method of the nitinol alloys, as well as the magnitude and rate of loading.

Finite element analysis was performed using the ABAQUS/Standard solver with the UMAT subroutine defined by individual user [40]. The nitinol properties for the UMAT inputs were referred from the paper published by Pham et al. [41]. Its ultimate strain and material endurance limit for zero mean strain were 10% and 1%, respectively, as reported by Lin et al. [14]. It was found that nitinol was unable to recover due to plastic deformation when the strain exceeded 10%. Therefore, 10% strain was used as a conservative upper limit in our study to minimize the potential risk of acute stent fractures.

#### 2.2.2. Loading and Boundary Conditions

**Figure 4:**
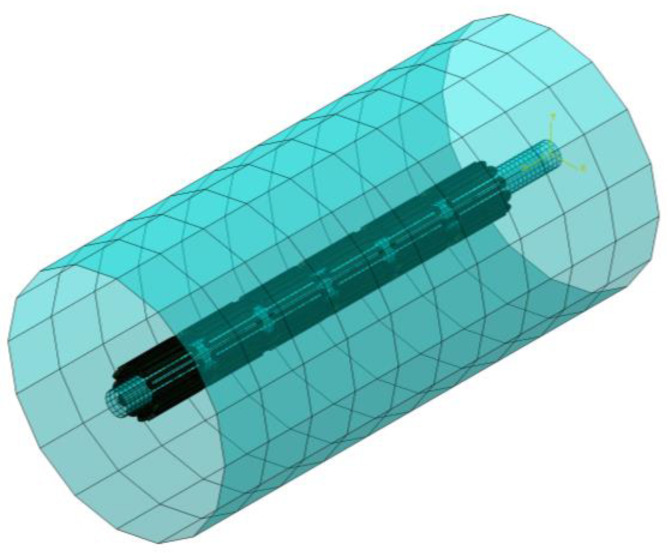
Assembly of finite element stent model with inner and outer cylinders.

Two cylinders (1 mm and 10 mm) were installed to our FEA model, with one cylinder placed inside the stent and the other cylinder outside the stent (Figure 4). During expansion, the inner cylinder was dilated to the pre-determined size and the resulting stresses/strains in all elements of the model were re-set to zero to simulate the relief of residual stresses during heat treatment. After that, the stent deformed geometry was saved and extracted for the next expansion. This process was repeated several times until the target diameter was reached (two times in our case). To simulate crimping, the outer cylinder was applied to compress and limit the stent movement inside a 2 mm catheter. A third cylinder of 5 mm was added to the model to imitate the inner wall of the artery. When the constraint from the outer cylinder was lifted, the stent recovered back to its original configuration until it hit the surface of the third cylinder and then stopped the recovery process. The stent was in full contact with the third cylinder for the fatigue life analysis.

The stent was meshed with the incompatible mode C3D8I element which is suitable for modeling objects that experience bending. These elements are fully integrated with added internal degrees of freedom, eliminating the shear locking phenomena. The above cylinders were meshed with the SFM3D4R element which has membrane and bending capabilities. This is a reduced integration element, meaning that it uses fewer integration points than the full integration element and provides much faster solutions, but may have some limitations in accuracy. The total element number was approximately 120,000 for the entire stent assembly. A frictionless contact condition was implemented to prevent mutual surface penetration with two contact pairs considered. Contact pairs are typically used to model the interactions between two surfaces that are in contact. The entire FEA simulation procedures were performed with five major steps:

1. First stent expansion to 4 mm ID (Inner Diameter), followed by heat treatment.
2. Second stent expansion to 6 mm ID, followed by heat treatment.
3. Stent crimping inside the 2 mm ID catheter.
4. Stent deployment into the 5 mm ID blood vessel.
5. Stent fatigue life assessment by oscillating ±3% stent diameter (4.85~5.15 mm, deviated from 5 mm norm).

Literature survey shows that the degree of the stent diameter oscillation varies in different clinical cases, including ±2.8% for the femoral artery [42], ±2.9% for the carotid artery [43], and ±1.7% for another femoral artery [44]. It was determined to apply ±3.0%, the maximum among all, to be the clinically worst-case scenario for investigation.

#### 2.2.3. Fatigue Life Analysis

The fatigue life of peripheral stents is a critical factor in their design and performance, as it could have a huge impact on patient safety and the success of the clinical outcome. Goodman fatigue life analysis has been widely accepted and provides a simple way to determine the fatigue life of a medical device or stent subjected to cyclic loading. Medical device companies typically perform extensive in vitro testing to evaluate the fatigue life of their products and ensure they meet the regulatory standards and requirements by Food and Drug Administration (FDA) [45].

Modified strain-based Goodman fatigue analysis has been adopted for nitinol stents in the past [14,46]. Following the FEA simulations, ±3% stent diameter (4.85~5.15 mm) corresponding to the systolic and diastolic pressure cycles was oscillated to simulate the pulsatile loading. The maximum and minimum values of the principal strains $\varepsilon_{1}$, $\varepsilon_{2}$, $\varepsilon_{3}$ due to the pressure cycles were collected at each integration point. They were used to calculate the principal mean strains $\varepsilon_{im}$ and principal strain amplitudes $\varepsilon_{ia}$, from which the effective mean strain $\varepsilon_{m}$ and the effective strain amplitude $\varepsilon_{a}$ were then obtained at each integration point:

(1) $$\varepsilon_{m}=\frac{1}{\sqrt{2}}\sqrt{{\left(\varepsilon_{1m}-\varepsilon_{2m}\right)}^{2}+{\left(\varepsilon_{2m}-\varepsilon_{3m}\right)}^{2}+{\left(\varepsilon_{3m}-\varepsilon_{1m}\right)}^{2}}$$

(2) $$\varepsilon_{a}=\frac{1}{\sqrt{2}}\sqrt{{\left(\varepsilon_{1a}-\varepsilon_{2a}\right)}^{2}+{\left(\varepsilon_{2a}-\varepsilon_{3a}\right)}^{2\;}+{\left(\varepsilon_{3a}-\varepsilon_{1a}\right)}^{2}}$$

Goodman equation is represented by a linear curve of mean vs. alternating strains that provides the information of device pass or fail. Modified strain-based Goodman life analysis indicates that stent fatigue failure could occur if the strain state in a stent satisfies the equation:

(3) $$\left(\frac{\varepsilon_{a}}{\varepsilon_{e}}\right)+\left(\frac{\varepsilon_{m}}{\varepsilon_{u}}\right)\geq 1$$

where $\varepsilon_{e}$ is the modified material endurance limit and $\varepsilon_{u}$ is the material ultimate strain. The modified material endurance limit $\varepsilon_{e}$ is defined as:

(4) $$\varepsilon_{e}=\varepsilon_{e0}\left(1-\frac{\varepsilon_{m}}{\varepsilon_{u}}\right)$$

where $\varepsilon_{e0}$ is the material endurance limit for zero mean strain. The material ultimate strain and material endurance limit for zero mean strain were 10% and 1%, respectively, as reported by Lin et al. [14].

The Goodman diagram is a graphical representation of the relationship between the normalized strain amplitude $\varepsilon_{a}∕\varepsilon_{e}$ and the normalized mean strain $\varepsilon_{m}∕\varepsilon_{u}$. The fatigue safety factor (FSF) is defined as the ratio of the modified material endurance limit divided by the strain amplitude. The purpose is to ensure that the predicted fatigue life of a stent exceeds its actual service life, even under the most severe loading conditions. If the FSF is less than 1.0, the stent fatigue failure may occur due to cyclic loading. On the other hand, the stent becomes more fatigue-resistant as the FSF increases to greater than 1.0.

(5) $$FSF=\frac{\varepsilon_{e}}{\varepsilon_{a}}$$

### 2.3. Stent Laser-Cutting

**Figure 5:**
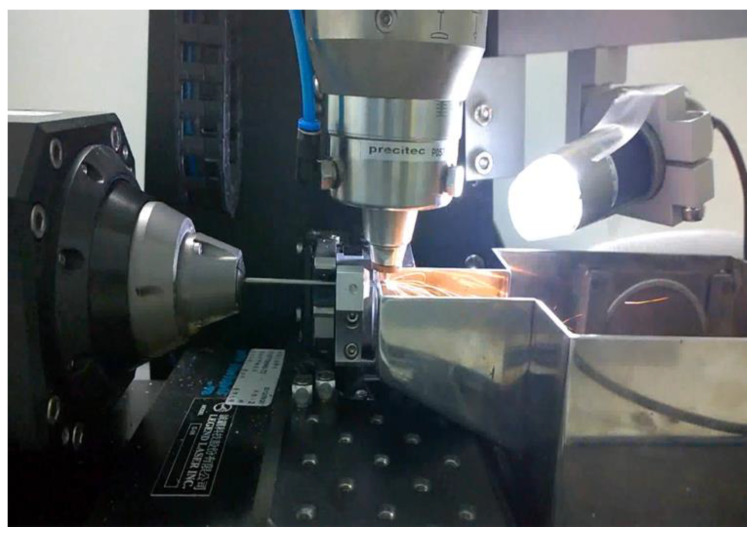
Stent design pattern cut onto a 2.0 mm hypotube by laser-cutting system.

A laser-cutting system included a Rofin StarFiber 180 FC Yb-doted CW laser (Rofin-Baasel Lasertech, Gilching, Germany), a linear motor stage (Aerotech, Inc., Pittsburgh, PA, USA), and a server motor were assembled (Figure 5). The laser has the power range of 10~100 W, wavelength of 1070 ± 10 nm, and pulse repetition rate of 170 kHz. The Aerotech linear motor stage has the horizontal travel distance of 100 mm and continuous rotational motions, with the maximum travel speed of 300 mm/s and maximum revolving speed of 600 rpm. It uses high-resolution encoders, high-torque motors, and advanced algorithms for achieving high accuracy. The linear movement and rotation of the hypotube were powered by the linear motor stage, whereas the vertical distance between the laser and target surface was controlled by the server motor to reach the optimal laser focal depth [47]. The stent design was laser-cut onto a 2.0 mm hypotube based on the coded geometry converted from CAD/CAM. Position synchronized output was implemented to precisely coordinate the motion of the linear motor stage with the timing of laser-firing, which greatly improved the accuracy and reliability of the entire system.

### 2.4. Heat Treatment and Surface Finishing

Nitinol heat treatment is critical in the production of nitinol-based devices, such as stents, orthodontic wires, and actuators. The heat treatment involves heating nitinol to a specific temperature for a certain period of time, followed by cooling it at a controlled rate. It is important to carefully control this process to ensure that the stent has the desired properties such as sufficient strength and flexibility, as small variations in temperature or cooling rate could affect the final properties of the nitinol devices.

To create the super-elastic effects, specific heat treatment process along with molding fixtures, were used after cutting to remove residual stresses and shape the device to the target size. Nitinol stents were progressively expanded by inserting rods of increasing sizes and heat-treated in a salt bath at an elevated temperature of 500 °C for 200 s. After each expansion, they were removed from the salt bath and quenched in cold water, ready for the next expansion [48].

The elevated temperatures from laser-cutting and heat treatment create spatter, oxide layers, and burn marks that need be removed by surface finishing. It was completed in two phases, first sand-blasting and then electro-polishing. Sand-blasting was carried out by spraying aluminum oxide particles of 28~32 μm at a pressure of 2 atm for 3 min to remove large debris. Electro-polishing was carried out with the solution of 79 vol% of acetic acid (CH_3_COOH, 99.5%) and 21 vol% of perchloric acid (HClO_3_, 70~72%). It was conducted at room temperature, with the first step at 7.7 volts for 60 s and the second step at 11.3 volts for another 60 s.

### 2.5. Rotating Bending Fatigue Testing

**Figure 6:**
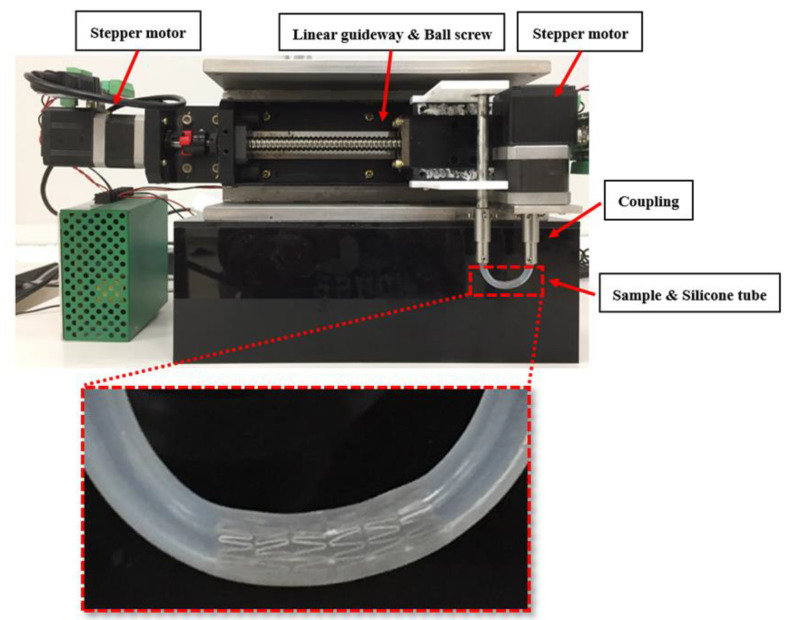
Rotating bending fatigue tester for a transparent tube with a stent deployed inside.

Our rotating bending fatigue tester consisted of several parts: three-phase stepper motors, a linear bearing module, a specimen holder, and RS232 for serial communication (Figure 6). The three-phase stepper motor was able to generate the tube rotation at a speed of up to 1000 rpm, whereas the linear bearing module controlled the tube arc formation. The stent was deployed inside the cylindrical tube, and the tube was then mounted onto the specimen holder (coupling). The motor rotated the coupling, and the cyclic bending loads were applied to the stent inside the tube. A 3 mm ID and 4.7 mm long transparent silicone tube was selected as the carrier for the tested stent. The span of the two couplings was 3 cm apart which was translated into an arc radius of 1.5 cm. Tests were manually interrupted at about every 1000 cycles for inspection of any stent fractures using an optical microscope. Body temperature was reached by immersing the stent-deployed tube in a fluid bath of PBS buffer solution at 37 °C.

## 3. Results

A simple yet powerful design concept to improve the stent fatigue life was studied. To evaluate the degree of fatigue enhancement, the midpoint of the strut was tapered from 100% to 60% of its original width (0~40% width reduction) in 10% increments for investigation. Fatigue safety factors were calculated and plotted as a function of the strut tapered degree. Stent samples were laser-cut, heat-treated, sand-blasted, and then electro-polished by various techniques mentioned previously. Rotating bending fatigue tests were conducted to validate the tapered-strut design concept.

### 3.1. Standard Stent Design

A standard stent design with a constant strut width throughout the stent was first investigated to establish the baseline information for the subsequent comparison. FEA simulations were performed on this standard stent design for the maximum strains at various stages of expansion/crimping/deployment and fatigue life analysis subjected to pressure cycles of ± 3%. Their respective values at various stages were 4.23% (expansion), 5.55% (crimping), 1.82% (deployment), and 1.11 (FSF).

**Figure 7:**
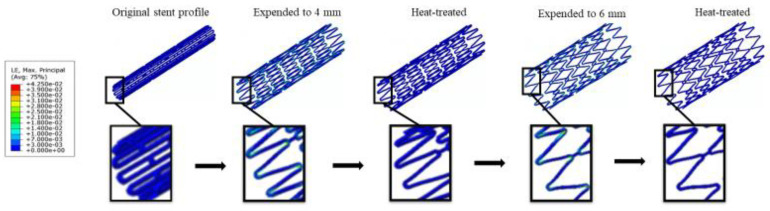
Contour plot comparison of strain distribution during multiple expansions and corresponding heat treatments for the standard design.

Figure 7 represents a series of contour plot comparison of the strain distribution during multiple expansions and their corresponding heat treatments for the standard design. At each expansion, the maximum strain always occurred at the inner surface of the crown, while there were only little strains on the stent strut connecting two crowns. The heat treatment following each expansion relieved all residual stresses, allowing the entire stent to return to its original stress-free status. The stent deformed geometry was saved and extracted for the next expansion repeatedly.

**Figure 8:**
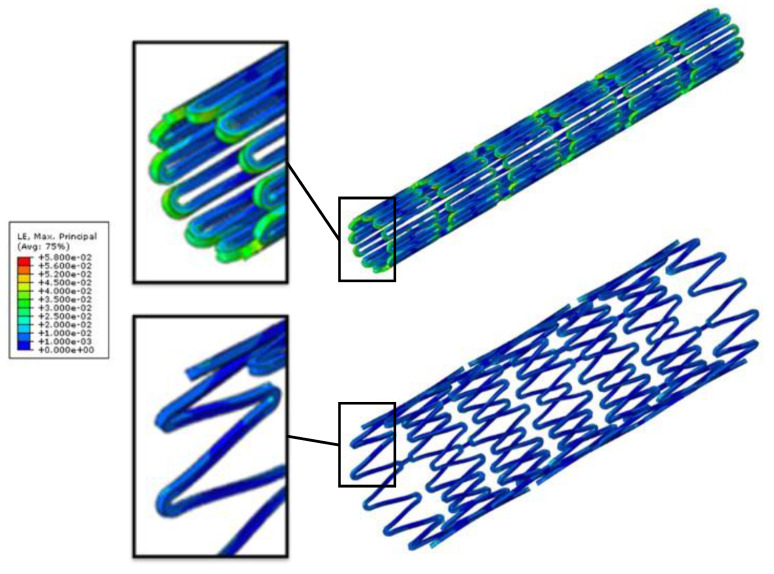
Contour plot comparison of strain distribution at crimping (**top**) and deployment (**bottom**) for the standard design.

In the next step, the stent was crimped down to the diameter of 2 mm and constrained inside a catheter delivery system. It was released from the catheter to allow for recovery and finally stopped when contacting the 5 mm arterial wall. Figure 8 shows the contour plot comparison of the strain distribution at crimping and deployment (released to the artery) for the standard design. As a last step, fatigue safety factor was evaluated using Goodman fatigue life analysis. Among all simulation steps, the crimping was the most critical step among all, as the maximum strain of 5.55% was reached. It should be noted that the maximum strain migrated from the inner to the outer surface of the crown during crimping, as shown in Figure 8.

### 3.2. Tapered-Strut Stent Design

A novel tapered-strut design concept to improve the stent fatigue life was studied. This tapered-strut design was exactly identical to the standard design in all dimensions for counterpart comparison, except that its strut width was tapered. The midpoint of the strut was tapered in 10% increments. FEA simulations were performed on both standard design and tapered-strut design for side-by-side comparison. Table 1 summarizes the FEA simulation results of the maximum strains at various stages and the fatigue safety factor as a function of the strut tapered degree. It shows that the maximum strains at each stage decreased progressively as the strut tapered degree increased. For example, at the most critical stage of the crimping process, the maximum strain dropped by 31% (from 5.55% to 3.84%) when the strut width was tapered by 40%.

**Figure 9:**
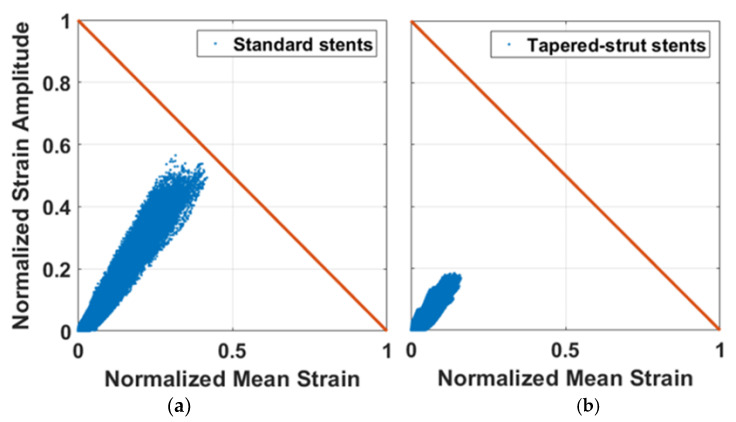
Goodman life analysis of a standard design (**a**) and a 40% tapered-strut design (**b**).

**Figure 10:**
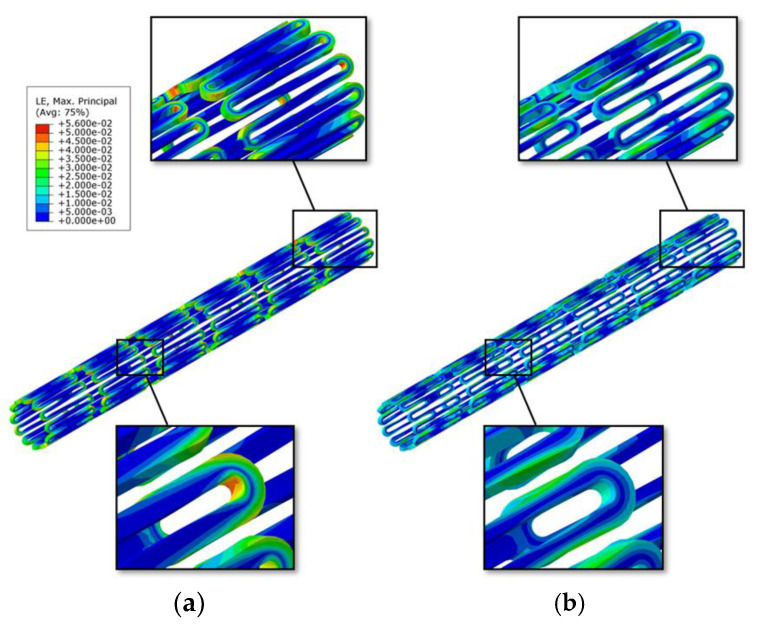
Contour plot comparison of strain distribution between a standard design (**a**) and a 40% tapered-strut design (**b**).

Figure 9 shows the Goodman diagrams for both standard design and 40% tapered-strut design. The diagonal Goodman failure line was used to predict the fatigue life under cyclic loadings. If the strain amplitude and mean strain fell below the Goodman line, then the stent would not fail due to fatigue. As shown, the tapered-strut design was far away from the failure line, indicating less prone to fatigue failure when compared to its standard counterpart. For the tapered-strut design with 40% taper, its FSF jumped by 4.2 times that of the standard design. It is surprising that such an enormous impact was produced by an inconspicuous design tweak. This amazing outcome could be attributed to the stress re-distribution after tapering the strut width, effectively moving the stress concentration away from the crown and re-distributing the stresses along the struts. In Figure 10, the red and yellow colors of the contour plot extended deeper into the mid-strut sections of the 40% tapered-strut design. Its strain distribution spread out more uniformly throughout the stent than that of the standard design, with reduced intensity level at the crown. This is a very efficient way to distribute energy by subjecting a larger volume of the stent to share the loadings.

**Figure 11:**
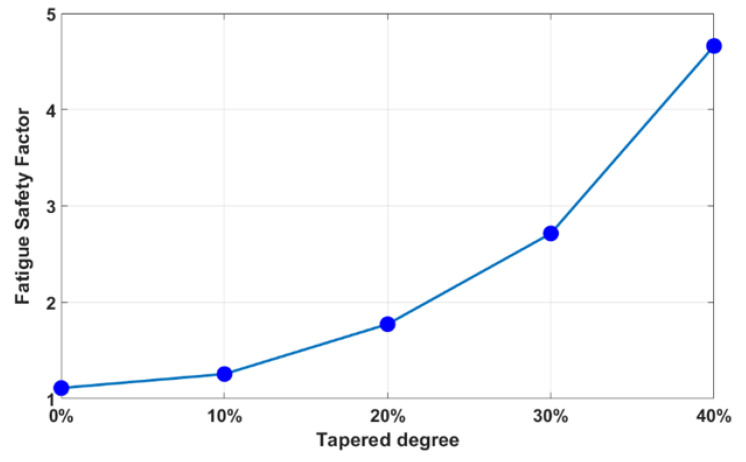
Fatigue safety factor as a function of the strut-tapered degree.

Figure 11 shows the fatigue safety factor as a function of the strut tapered degree in 10% increments. The fatigue safety factor jumped sharply with a few folds improvement as the strut tapered degree increased, especially around 20~40%. A 40% taper significantly enhanced the FSF by 4.2 times, while a 20% taper led to a smaller gain of 1.6 times.

To validate the tapered-strut design concept and its effects in stress re-distribution, bench fatigue tests were conducted on stent samples made in-house at room temperature of 23 °C and body temperature of 37 °C, using the built rotating bending fatigue tester. Body temperature was reached by immersing the stent-deployed tube in a fluid bath located at the bottom of the fatigue tester. Both standard stent and their tapered-strut counterpart (40% taper) were tested under the same loading conditions for comparison, with the sample size of at least five for each category. A total of thirty samples were made, with twelve standard stents and eighteen 40% tapered-strut stents. They were further divided into two testing groups of room temperature and body temperature. Among them, two samples were fractured during manufacturing at expansion so twenty-eight samples were tested in total. Fatigue tests were manually interrupted at about every 1000 cycles for inspection using an optical microscope. The fatigue cycle number was recorded when the first stent fracture was captured.

Fatigue life of a stent depends on various parameters such as material properties, stent design, and manufacturing, as well as test operating and loading conditions. In order to evaluate the tapered-strut effects, all factors were made identical except the stent design. Table 2 shows the fatigue cycle number at fracture from rotating bending fatigue tests. At room temperature, the average cycle number of the standard design was 7187 cycles, while that of the 40% tapered-strut design jumped to 47,713 cycles with a gain of 6.6 times. At body temperature, the average cycle number of the standard design was 8009 cycles, while that of the 40% tapered-strut design was 47,567 circles with a similar gain of 5.9 times. This jump in the stent fatigue life was significant and validated the increasing trend predicted by FEA.

## 4. Discussion

A stent is a small, mesh-like tube that is implanted into an artery to help keep it open and improve the blood flow. An ideal stent has enough radial strength to provide support to the artery, good flexibility to be easily maneuvered during surgery, and low profile to reduce the risk of injury when delivered inside the body. Often, these clinical attributes compete with one another such that enhancement in one attribute usually leads to the trade-off in others. Therefore, stent design is a balance of art and need to be taken care of cautiously. The choice of stent design depends on the specific patient needs, including the location and severity of the blockage, as well as the patient’s overall health conditions.

When a stent is crimped or deployed, the maximum stress is typically observed on the crown, particularly at its edges and corners. In contrast, there are very little stresses developed on the strut, which connects two opposing crowns to form a zigzag pattern. Therefore, design modifications directly on the stress-free strut could enhance the stent fatigue life, with minimal impact on other properties. Our tapered-strut design concept is to move the stress concentration away from the crown and re-distribute the stresses along the strut by tapering the strut width at the midpoint. This slight design tweak is able to reduce the loading burden on the crown, allow more efficient energy distribution, and thus significantly improve the stent fatigue life.

The integration of FEA analysis and bench testing results in a much faster and better evaluation process for the development of modern medical devices. Fatigue testing is an important aspect of stent design and assessment, as it provides valuable information on the mechanical integrity and durability of stents, and can help to identify potential design issues that may lead to stent fatigue failure in vivo. Standard stent fatigue testing is typically performed via ASTM F2477 “Standard Test Methods for in-vitro Pulsatile Durability Testing of Vascular Stents”, which involves placing stents into mock arteries and subjecting them to pulsatile loadings of at least 400 million cycles under controlled environment. This method specifies the use of a stent crimping device to simulate the implantation, and a custom-designed test apparatus (such as Bose ElectroForce Fatigue Test Instrument) to apply the cyclic loadings. The test is typically performed in a saline solution to simulate physiological conditions. Fatigue testing can either be performed by pressure-control or displacement-control to reproduce the expansion and contraction that a stent experiences in vivo. They are performed at frequencies of up to 30~60 Hz, resulting in an accelerated test duration of 3~6 months. The FDA acceptance criterion is a simple pass/fail test for success, in which no stent fractures should occur during fatigue testing. Another test method being adopted is the “Fatigue to Fracture” approach. It involves a combination of FEA analysis and bench fatigue testing to evaluate the stent durability through developed theories or techniques such as fracture mechanics [49].

Rotating bending fatigue testing emerges as another method of testing stent fatigue performance. Eggeler and other researchers did a great deal of work on nitinol wires using this test method [50,51,52,53]. This type of tester applies cyclic bending to a wire while rotating it around an axis, creating the cyclic loading conditions based on the testing needs. To quickly prove our tapered-strut design concept, a rotating bending fatigue tester was built in-house, with a stent-deployed tube being tested instead of a wire. This fatigue tester was based on the rotating beam theory by looping a stent through a pre-determined tube arc, from which the bending stresses in the stent were determined. When half revolution was rotated, the stresses above the neutral axis were reversed from compression to tension, and vice versa. When completing one revolution, the stent experienced a complete cycle of combined tension and compression. This harsh environment for stents made it possible to test a large number of samples within a short time to quickly collect the fatigue data for a given tube arc (cyclic stress level). Unlike ASTM F2477, this test measures the number of cycles that a stent can withstand before fatigue failure, and provides an efficient means of comparing the fatigue performance of different stent designs or stent materials.

**Figure 12:**
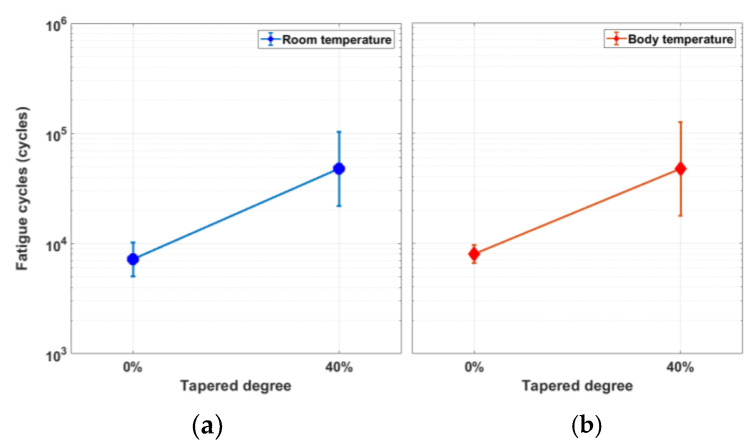
Fatigue cycle number comparison between standard design and 40% tapered-strut design at room temperature (**a**) and body temperature (**b**).

The effects of the tapered-strut design on the fatigue life enhancement of peripheral stents were investigated numerically and experimentally. In order to evaluate the fatigue enhancement, the stent strut was tapered from 100% to 60% of its original width in 10% increments. Fatigue safety factors were calculated using FEA and plotted as a function of the strut tapered degree. Rotating bending fatigue tests were conducted to validate the tapered-strut design concept. There were two outputs used to measure the correlation between FEA simulation and actual testing: fatigue safety factor (FEA) vs. fatigue cycle number (testing) and maximum strain location (FEA) vs. stent fracture location (testing). FEA simulation results show that the FSF increased sharply as the strut width was tapered. A 40% taper enhanced the FSF by 4.2 times. This FEA prediction was further validated by rotating bending fatigue tests, with 6.6- and 5.9-times fatigue life enhancement at room temperature of 23 °C and body temperature of 37 °C, respectively. In Figure 12, the measured fatigue cycle numbers show similar trends in both cases, suggesting that temperature does not affect the stent fatigue life, at least within the temperature of our interests.

**Figure 13:**
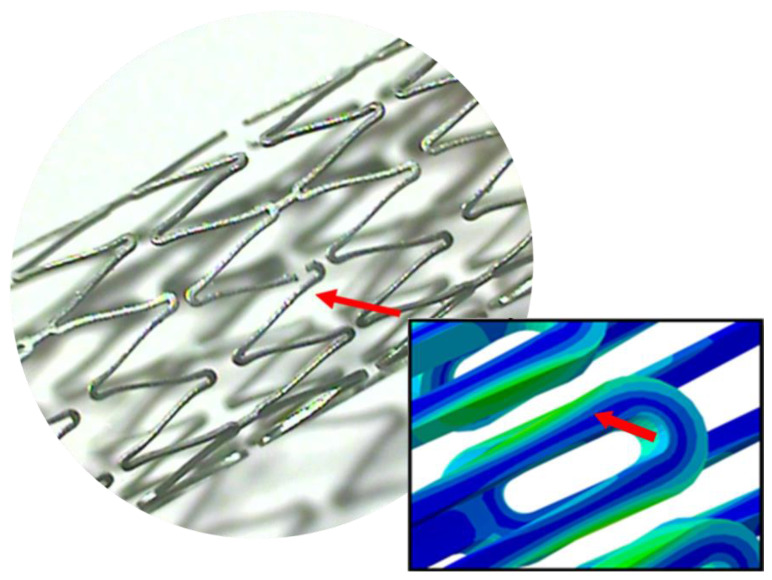
Stent fracture after the rotating bending fatigue testing.

Stent laser-cutting is a key step in the manufacturing of stents, as it provides precise and controlled laser-cuts with minimal heat damages to the surrounding material. This makes it possible to produce stents with intricate designs that would be difficult to achieve using traditional methods. It was observed that testing data from the 40% tapered-strut design were more scattered than those of the standard design. This could be attributed to the laser-cutting issues, as cutting the curved lines on a stent with precision was more challenging than the straight lines. The laser-cutting quality is dominated by key factors including laser focal position and pulse repetition rate. The laser focal position refers to the point in space where the laser beam is focused to achieve the desired intensity and precision on an object. The position of the focal point can be adjusted by changing the vertical distance between the laser source and the object or by changing the shape of the focusing lens. An appropriate laser focal position significantly improves the laser-cutting quality. The pulse repetition rate refers to the number of laser pulses that are emitted by the laser at a given period of time. Typically, a higher repetition rate can result in faster cutting speeds and higher throughput, but it can also increase the heat input and reduce the quality of the cut. In contrast, a lower repetition rate can result in slower cutting speeds but may improve the cutting quality by reducing heat input and minimizing the risk of thermal damage to the stent. Figure 13 shows the stent fracture after the rotating bending fatigue testing was stopped. The FEA strain contour plot of the stent strut was also added next to the photomicrograph for side-by-side comparison of the stent fracture location vs. the maximum strain location. It was noted that FEA simulation and actual testing agreed very well to each other, with the stent fracture occurring at the hinge point between the strut and crown, as indicated by the red arrows. This location had the maximum stress after the stress re-distribution due to the taper-strut effects. Coincidently, this was also the location where the design curvature changed from the convex to concave shape, which could cause some hiccups during laser-cutting if not setting up properly. This may explain why the fatigue cycle numbers of the 40% tapered-strut design were measured as low as 8149 cycles but as high as 184,799 cycles in Table 2. Given the variability, the fatigue cycle numbers of the 40% tapered-strut design still far exceeded those of the standard design by multiple times regardless. Due to the fairly large variance of fatigue cycle numbers from bench testing, a statistical analysis on the significance of difference were performed to further demonstrate the definite benefits of our proposed design.

A one-tailed *t*-test was performed to determine if there was a statistically significant difference between the means of two variables for our case comparison (standard design vs. 40% tapered strut design; room temperature vs. body temperature). Like most other tests of significance, the threshold in a *t*-test is typically set at *p* = 0.05. If a *p*-value from a *t*-test is less than 0.05, then the result is considered to be statistically significant. Table 3 lists the calculated *p*-values from *t*-tests when evaluating the tapered-strut effect and the temperature effect, respectively. When the temperature was maintained at 23 °C, there was a statistically significant difference (*p* = 0.017) between the standard design and the 40% tapered-strut design. Similar result was also obtained for the case of 37 °C, which showed statistically significant difference (*p* = 0.036) between the two designs. On the other hand, when the stent design was made identical, there was no statistically significant difference (*p* = 0.298 for the standard design and 0.498 for the 40% tapered-strut design) between two temperatures, suggesting again that temperature does not affect the stent fatigue life.

**Figure 14:**
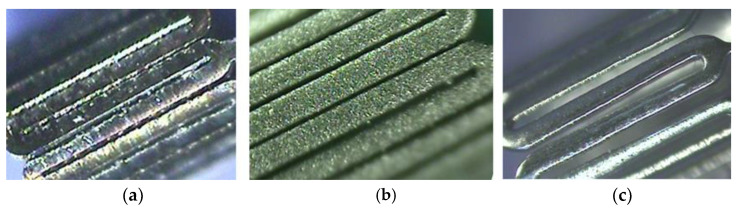
A stent after laser-cutting (**a**), sand-blasting (**b**), and electro-polishing (**c**).

Two tested stents were fractured during manufacturing at expansion. In addition to the laser-cutting issues, other factors such as heat treatment and surface polishing could cause stent fractures during manufacturing as well. Figure 14 shows a series of pictures after laser-cutting (left), sand-blasting (middle), and electro-polishing (right) procedures. The surface was sand-blasted and then electro-polished to achieve a mirror-like finishing. Heat treatment was used to reduce residual stresses by subjecting the stent to a specific temperature range for a certain period of time, followed by controlled cooling. This could cause the material to undergo a phase transformation or re-crystallization, reducing the magnitude of residual stresses; however, its effectiveness depends on the specific material and the initial stress level as well. During heat treatment, temperature and time were found to be the most critical parameters in manufacturing. Inappropriate temperature and operating time impacted the stent performance negatively.

## 5. Conclusions

A simple yet powerful tapered-strut design for enhancing the stent fatigue life was studied. The key feature of this design is to move the stress concentration away from the crown, forcing the stress-free strut to share more loadings by tapering its strut geometry. FEA simulation results show that the FSF of the 40% tapered-strut design jumped sharply by 4~5 times that of its standard counterpart. A rotating bending fatigue tester was built in-house and bench fatigue tests were conducted. A total of thirty samples were made, with twelve standard stents and eighteen 40% tapered-strut stents tested at room temperature and body temperature. Bench test results show that the 40% tapered-strut design successfully increased the fatigue life by 6~7 times that of a standard design, which agreed very well with the increasing trend in FSF predicted by FEA. This design concept has a great potential in fatigue enhancement and points to an important direction for future stent designs to follow.

## Figures and Tables

**Table 1:** Maximum strains and fatigue safety factor as a function of the strut tapered degree.

	Expanded to 4 mm	Expanded to 6 mm	Crimped to 2 mm	Released to 5 mm	Fatigue Safety Factor
Standard Stent	3.90%	4.23%	5.55%	1.82%	1.11
90% Width	3.44%	3.73%	4.94%	1.52%	1.26
80% Width	2.92%	3.35%	4.30%	1.35%	1.77
70% Width	2.41%	2.82%	3.91%	1.22%	2.72
60% Width	1.88%	2.31%	3.84%	1.20%	4.66

**Table 2:** Fatigue cycle number at fracture from rotating bending fatigue tests.

Sample	Standard (23 °C)	40%Taper (23 °C)	Standard (37 °C)	40%Taper (37 °C)
1	6464	21,022	10,770	93,471
2	11,681	66,938	8634	8149
3	8243	147,926	8447	10,709
4	4175	38,717	6697	24,442
5	5374	19,002	7268	184,799
6	N/A ^1^	21,323	6243	30,311
7		26,120		34,078
8		40,657		21,949
9		N/A^1^		20,197
Average	7187	47,713	8009	47,567
SD	2615	40,685	1505	54,028

^1^ Stent fractures during manufacturing at expansion.

**Table 3:** One-tailed t-test for evaluation of tapered-strut and temperature effects.

Control	23 °C	37 °C	Standard	40% Taper
Test	Standard vs. 40% Taper		23 °C vs. 37 °C	
*p*-value	0.017	0.036	0.298	0.498

## Data Availability

The data used to support the findings of this study are included within the article.
